# Identifying tweets of personal health experience through word embedding and LSTM neural network

**DOI:** 10.1186/s12859-018-2198-y

**Published:** 2018-06-13

**Authors:** Keyuan Jiang, Shichao Feng, Qunhao Song, Ricardo A. Calix, Matrika Gupta, Gordon R. Bernard

**Affiliations:** 1 Department of Computer Information Technology and Graphics, Purdue University Northwest, Hammond, IN USA; 2 Department of Medicine, Vanderbilt University, Nashville, TN USA

**Keywords:** Health surveillance, Pharmacovigilance, Social media, Twitter, Deep learning, Unsupervised feature learning, LSTM neural network

## Abstract

**Background:**

As Twitter has become an active data source for health surveillance research, it is important that efficient and effective methods are developed to identify tweets related to personal health experience. Conventional classification algorithms rely on features engineered by human domain experts, and engineering such features is a challenging task and requires much human intelligence. The resultant features may not be optimal for the classification problem, and can make it challenging for conventional classifiers to correctly predict personal experience tweets (PETs) due to the various ways to express and/or describe personal experience in tweets. In this study, we developed a method that combines word embedding and long short-term memory (LSTM) model without the need to engineer any specific features. Through word embedding, tweet texts were represented as dense vectors which in turn were fed to the LSTM neural network as sequences.

**Results:**

Statistical analyses of the results of 10-fold cross-validations of our method and conventional methods indicate that there exist significant differences (*p* < 0.01) in performance measures of accuracy, precision, recall, F1-score, and ROC/AUC, demonstrating that our approach outperforms the conventional methods in identifying PETs.

**Conclusion:**

We presented an efficient and effective method of identifying health-related personal experience tweets by combining word embedding and an LSTM neural network. It is conceivable that our method can help accelerate and scale up analyzing textual data of social media for health surveillance purposes, because of no need for the laborious and costly process of engineering features.

## Background

Social media have naturally become an active source of health surveillance data because of their wide availability and easy accessibility for users to share their personal health experience freely online. Applications of social media data for health surveillance have been investigated in the areas of disease surveillance and outbreak management [1], illicit drug uses [2] and pharmacovigilance [3, 4]. Among various data sources, Twitter, in particular, has attracted a lot of interests as a data source for health surveillance activities such as forecasting influenza [5], drug safety surveillance [6, 7], and detection of potential effects of using dietary supplements [8]. Twitter is a general purpose microblogging service, and in most of the published studies, Twitter posts collected are not necessarily health related, let alone the personal health experience posts.

Analyzing Twitter data poses special challenges to many aspects of natural language processing (NLP) and machine learning-based classification. Twitter data possess unique characteristics not found in other data. With the limitation of 140 characters, Twitter users have been creative in generating short texts that neither exist in dictionaries nor follow the grammatical and spelling rules. Conventional machine learning-based classification methods require features extracted from the raw data. The task of feature extraction is both science and art. Different kinds of data and classification will require different kinds of features. Identifying and determining which features are of merits to be included is a process requiring significant human intelligence, especially when raw data are in the format that can not be used directly as features.

There are two types of data in each Twitter post that can be used as features: textual data from the tweet text and metadata such as the date when a tweet was posted, the client application used to post the tweet, and the number of retweets. The metadata typically do not contain much rich semantic information and can be readily used as features if needed without any conversion or transformation. On other hand, the textual portion of a tweet can contain very rich linguistic information pertaining to the health issues being investigated. Irregular linguistic expressions contained in the tweet text make conventional natural language processing (NLP) techniques perform poorly by incorrectly tagging parts of speech (POS) and/or failing to recognize named entities. The incorrect information in the result of the NLP will lead to incorrect feature data, making the classifiers behave poorly.

Conventional NLP techniques and machine learning-based classification methods do not seem to perform well with Twitter data, and published results were mostly based upon small datasets which are not necessarily representable to the entire population of the Twitter data.

With the continuing availability of the significant amount of Twitter posts, it is important to develop more reliable and accurate classification methods to process and analyze Twitter data for study of health related issues. In this research, we presents a method developed based upon the word embedding and LSTM neural network to predict personal experience tweets (PET) pertaining to the use of pharmaceutical products.

## Method

Our approach first generates a term index vector space model (VSM) from the textual data of unlabeled tweet corpus, and each vector represents the index of a unique term or token in the tweet text. Afterwards, a sequence of vectors representing each study tweet is constructed using the VSM and fed to a long short-term memory (LSTM) neural network that performs binary classification: personal experience tweet (PET) or non-personal experience tweet (non-PET).

### Personal experience tweets

As defined in [9], personal experience pertains to a person’s encounters or observations related to his or her life, and it can be the changes experienced by an individual in his or her health, which can be related to an illness, a disease, or a medical treatment. Such experience has been considered important in using social media data for public health surveillance [10–14] and little has been done in developing computational methods that can identify personal health experience tweets. In their work to detect potential drug effects by mining Twitter data, Jiang and Zheng [15] recognized the importance of differentiating personal experience tweets (PETs) from other irrelevant tweets, and chose personal pronounces as features to distinguish personal experience tweets from others. Being able to predict PETs and non-PETs can not only help collect public health-related information from the relevant tweets, but can also eliminate much of irrelevant Twitter posts which can be product promotions, news articles, and even spam. Jiang and colleagues [9] engineered a set of 22 features from Twitter textual data and metadata to detect PETs with conventional classifiers such as decision tree and k-nearest neighbors. Their performance results were marginal for precision, which could be attributed to the features engineered.

Below are examples of the personal experience tweets recently posted pertaining to users’ experience with Aspirin.

*“Thank you aspirin. No more headache”.*

*“I have a headache in my chest, from all the chaos that you left, caffeine and Aspirin take me away”.*

*“Out of aspirin. Currently having a migraine.”*

As can be seen, these examples show the various ways of expressing users’ experience, and such variation makes it challenging in identifying the useful tweet textual features for predicting PETs correctly.

### Representation of tweet

Inspired by the recent advancement in achieving high accuracies in recognizing objects from images using raw image data along with deep neural networks [16, 17], we designed an approach that explores the usage of raw tweet textual data as input to the deep neural network-based classifier. Unlike image data, raw textual tweet data can not be directly represented as dense vectors, and the number of tokens in tweet text is of varying sizes, which is contrary to what conventional classifiers require. We would like to leverage distributed representations of word (or word embedding) to represent individual terms in the tweet text as dense vectors. To do so, we introduced two special items in the vocabulary which is the collection of unique terms in the tweet text: padding (“pad”) and unknown (“unk”) to achieve the fixed length input and represent any unseen tokens. However, these two special terms do not have the corresponding text expressions, and hence can not be represented as vectors. To solve this issue, we replace the terms of tweets in question with the indices of terms in the vocabulary, making text of each tweet a sequence of indices (positive integers), rather than a sequence of textual terms. Dense vectors can then be generated on the term index sequences and their representations are equivalent to the ones of the original tweet text.

For example, the term index form of tweet “Thank you aspirin. No more headache” may look like what is shown in Table 1.

**Table 1:** Representation of an example tweet

Tweet Text	Thank	you	aspirin	No	more	headache
Symbolic Term Index	*i*_*Thank*_	*i*_*you*_	*i*_*aspirin*_	*i*_*No*_	*i*_*more*_	*i*_*headache*_
Actual Term Index	5918	1012	720	3973	241	2354

In Table 1, the first row represents the sequence of terms (or tokens) in the tweet. The second row is the sequence of symbolic representation of indices (i_term_) of corresponding terms in the vocabulary and the last row lists the sequence of actual term indices whose values are determined by the term positions in the vocabulary.

It has shown that the distributed representation of words in vector space embeds rich syntactic and semantic information of the words [18, 19]. In our approach, we created a vector space model from the term index representation of tweets — that is, instead of using the original tweet texts, the index-term format of tweet texts was used to generate the dense vectors in the model. We hoped that such treatment would provide meaningful information embedded in each tweet to the classifiers. In implementation, word2vec was used to build the vector space model.

### The long short-term memory neural network

Identification of PETs can be considered a binary text classification problem. It is a classic topic for natural language processing, in which one needs to assign predefined categories to free-text documents [20].

Compared to conventional classifiers, deep neural networks (DNNs) have demonstrated better performance in classification problems [21–24], and have, in recent years, won numerous contests in pattern recognition and machine learning [25]. An improved model from DNN is the recurrent neural network (RNN) which analyzes the text word by word and stores the semantics of all the previous text in a fixed-sized hidden layer [26]. The main advantage of RNN is its ability to better capture the contextual information and this could be beneficial to capture semantics of text [27].

The unique characteristic of RNN to store the former semantics of text makes it a great classifier candidate for PET prediction. Studies have shown that the text classifiers using RNN or based on RNN performed better in accuracy and precision. Examples of such effort include using gated RNN for document-level sentiment classification [28], and sequential short-text classification based on recurrent and convolutional neural networks [29]. However, RNN is incapable of continuing learning from the previous information encountered as time elapses. In the text classification model, it means that the RNN cannot memorize the context which are 5–10 words far away from current one. To deal with this issue, researchers developed the long short-term memory (LSTM) model based on RNN, which adds a forget gate to learn solving complex long text issues [30]. The LSTM model has been applied to text classification problem such as the text classification based on the combination of convolutional and LSTM neural network [31].

In this study, we converted our classification into a sequence classification problem which can be dealt with properly by the LSTM model. The input to the LSTM classifier is the distributed representation of tweet text (word embedding). The classifier was first trained with a set of annotated tweets (training set) and later the trained model performed classification on another set of tweets (test set).

### Data processing and analysis pipelines

**Fig. 1:**
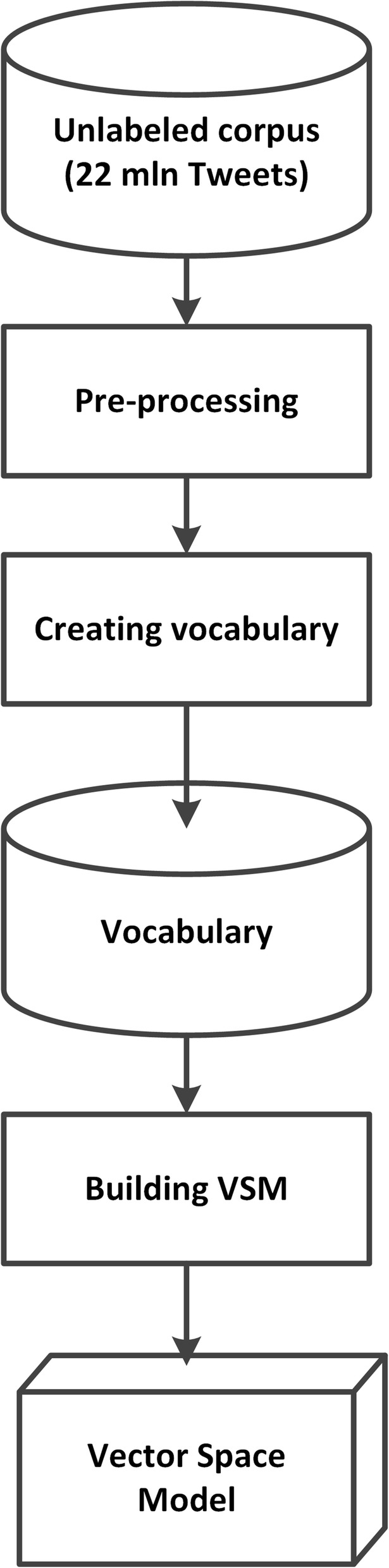
The pipeline to generate the vocabulary and vector space model. A corpus of 22 million unlabeled tweets was collected and pre-processed to remove certain punctuations, duplicates, non-English tweets, and tweets with URLs. A collection of unique terms was compiled to generate a vocabulary, and a vector space model was created the preprocessed tweets

Two separate pipelines were devised to (1) create the vocabulary and vector space model (VSM) from the unannotated tweets and (2) represent study tweets as sequences of dense vectors and classify the tweets with the LSTM neural network. The first pipeline shown in Fig. 1 is to create the vocabulary of the Twitter terms and build the vector space model (VSM). A corpus of a large quantity (22 million) of unlabeled tweets was first preprocessed to remove retweets and non-English tweets as well as performing phrase learning to identifying phrases which were then added to the vocabulary. A vector space model of the term index was generated, and was used in the second pipeline.

**Fig. 2:**
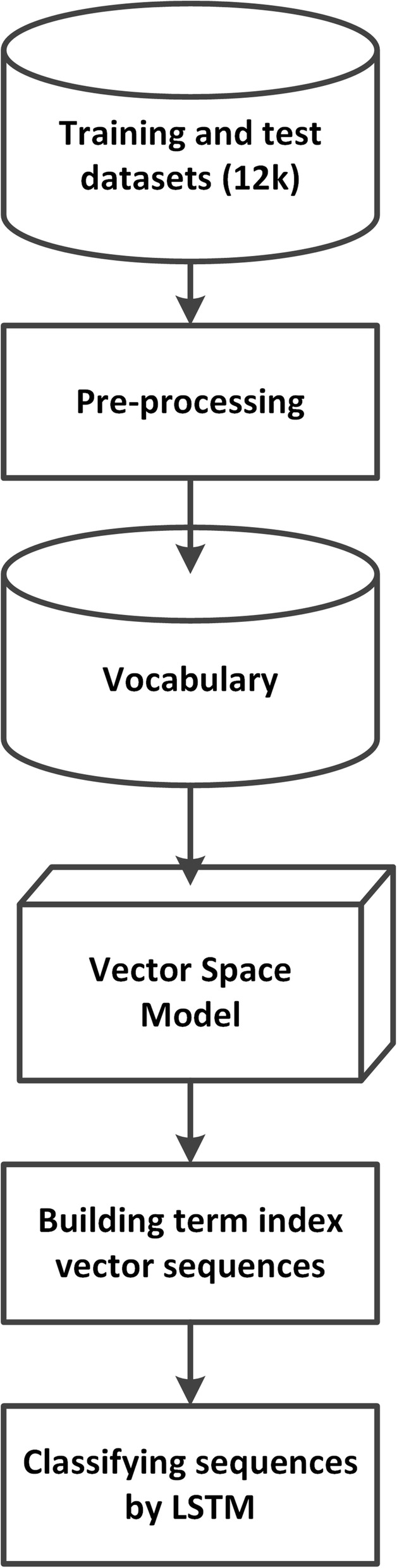
The pipeline to represent study tweets and classify the tweets. A total of 12,331 annotated tweets for training and test were preprocessed first. The index of each term in the preprocessed tweets was retrieved from the vocabulary, and the text of each tweet was converted to a sequence of the vectors of the corresponding term indices (see Fig. 3). Sequences of term index vectors were fed to the LSTM network for classification

The second pipeline illustrated in Fig. 2 shows the steps involved in representing each tweet in an annotated corpus (12,331 tweets) for training and testing as a sequence of term index vectors. It first determines the positions of tweet terms in the vocabulary, and using the position information (indices) locates the corresponding dense vectors, and arranges the vectors accordingly to form a sequence of term index vectors.

### Twitter data

A corpus of 22 million unlabeled tweets was used to build the vocabulary and vector space model. The tweets, containing the name of any of pre-selected 103 medicines, were collected using Twitter Streaming APIs from 25 August 2015 to 7 December 2016. To construct a corpus of annotated tweets, we employed an iterative method descripted in [9], and the resultant 12,331 tweets were randomly selected from the 22 million corpus and used for training and testing of classifiers. To annotate the tweets, a guideline of annotation was developed. The guideline defines what a personal experience tweet (PET) is and lists examples of PETs and non-PETs. Using the guideline, three annotators labelled independently a set of 100 tweets, and the annotation results were reviewed and revised by the first author and the annotators to establish the annotation gold standard. The same annotators completed the remaining tweets independently with the guideline and gold standard. Afterwards, another researcher (MG) stepped in as the disagreement resolver who settled the disagreed labels due to the subjectivity of human annotators and ambiguity of tweet text.

The same set of the annotated tweets was used for both conventional classifiers and our method. In the annotated tweets, retweets and non-English tweets were removed to eliminate duplicate information and facilitate downstream processing. The composition of the annotated tweet corpus is shown in Table 2. Tenfold cross-validation was used for all methods to facilitate validation and statistical analysis.

**Table 2:** Statistics of the corpus of annotated tweets

# of Tweets	# of PETs	# of Non-PETs
12,331	2962	9369

### Implementation

**Fig. 3:**
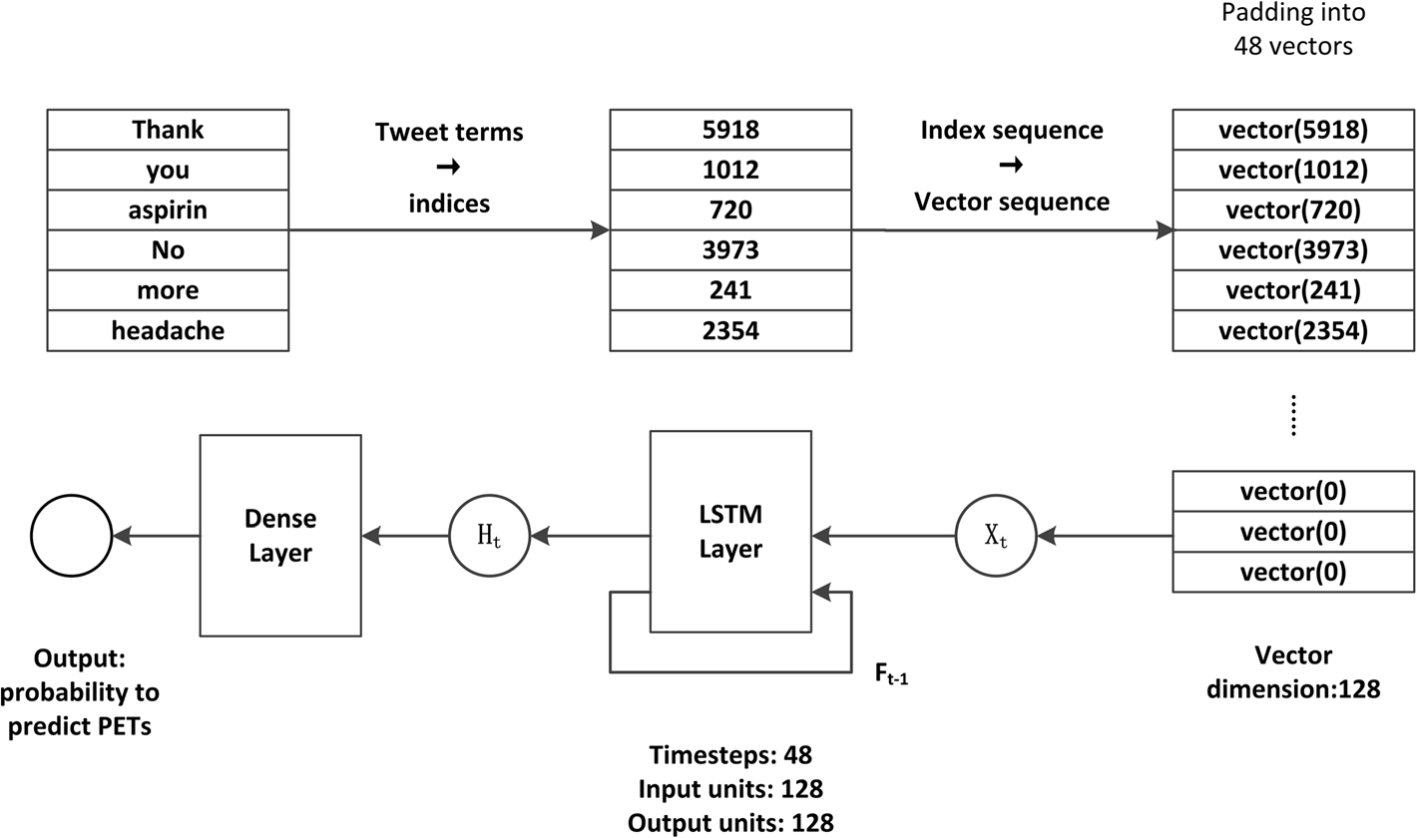
The high level overview of the LSTM model

The high level architecture of the LSTM model of this study is illustrated in Fig. 3. As can be seen, the text of each tweet is formatted as a sequence of 48 index term vectors — the number 48 was the largest number of tokens of the tweets we collected. If a tweet is shorter than 48 tokens, index/indices of “pad” will be appended to the sequence. Each token is represented as a 128 dimensional vector of the index of the corresponding term in the tweet text. Hence, for each tweet, a sequence 48 vectors of 128 dimensions was fed to the LSTM classifier. The LSTM model uses a set of transition functions to process the input sequence, and yields the output.

For this study, the LSTM model was based upon the implementation in Keras (https://keras.io/), a front-end for the combination of Google’s TensorFlow (https://www.tensorflow.org/) and Theano. We chose Tensorflow as the backend. The LSTM model was made up of three base layers: word embedding layer, LSTM layer and the final dense layer to receive the results.

Our model was trained with the training set over 200 epochs and the accuracy of the model was recorded in each epoch. We observed that the accuracy changes became stable around 5 epochs. Based upon our observation, we chose 5 epochs to train the model. In addition, this model used a general L2 regularization. The output units of the word embedding layer were 128 dimensional dense vectors, and there were 48 time steps in the LSTM layer in order to accommodate the tweets with the largest number of tokens. Finally, as this model was trained with the class-imbalanced training set, we also implemented the adjustment of class weight accordingly, in order to boost the importance of the minority class. The weight of the minority (PET) class was derived by the ratio of the number of majority class (non-PET) instances to the number of minority class (PET) instances.

## Results

To investigate the performance of our approach of combining word embedding and LSTM model, we used 4 conventional classifiers (logistic regression, decision tree, k-nearest neighbors, and support vector machine) as the baseline methods in comparison with our method. A set of 22 human-engineered features was used as the input for conventional methods and 128 dimensional term index vectors were used as the input for our method. The 22 engineered features were derived from linguistic characteristics and metadata of tweets, and they include POS tags, occurrences of commonly occurred tokens in one class of tweet text and user name but not in the opposite class, count of URLs, client application, and so forth [9]. In addition, we also included the result of using the bag-of-words (BoW) model with logistic regression. The BoW approach does not require engineered features either, but represents tweets as sparse vectors of occurrences of words. In our study, the dimension of the sparse vectors is 18,515. Summarized in Table 3 are the settings of parameters of the classifiers used in this study. All our methods were implemented using Scikit-learn library [32].

**Table 3:** Parameter settings of classifiers

Classifier	Parameter Settings
Logistic Regression	penalty:'l2’, tol = 1e-4, C = 1.0, solver:'liblinear’,max_iter = 100
Decision Tree (J48)	criterion = ‘entropy’, max_depth = 30, min_samples_split = 2, min_samples_leaf = 1
KNN	n_neighbors = 1, *p* = 2, metric = ‘minkowski’,algorithm = ‘auto’
SVM	C = 1.0, kernel = ‘rbf’, tol = 1e-4, gomma = 0.001
BoW + Logistic Regr.	C = 1000, random_state = 0
Word Embedding + LSTM	In LSTM layer, the input and output dimensions: 128, L2 for regularizer, and the parameter for L2: 0.01. 30% of training dataset was used as validation dataset. Class weight for PET class: 6547/2650, and for non-PET class: 2650/6547

Listed in Table 4 are the means of the performance measures from 10-fold cross-validations of all the methods tested in this study.

**Table 4:** Classification performance

Classifier	Accuracy	Precision (PET)	Recall (PET)	F1 (PET)	ROC/AUC
Logistic Regression	0.637	0.356	0.471	0.405	0.598
Decision Tree	0.602	0.329	0.442	0.357	0.547
KNN	0.669	0.383	0.481	0.411	0.604
SVM	0.635	0.339	0.478	0.393	0.580
BoW + Logistic Regr.	0.757	0.498	0.567	0.530	0.698
Word Embedding + LSTM	**0.815**	**0.598**	**0.702**	**0.645**	**0.776**

The highest values are in boldface

Each numeric value in Table 5 is the *p*-value of the one tail paired t test on the means of the corresponding performance measure (the column heading) between our method and the corresponding classification method (the row heading). This was intended to serve the purpose of evaluating the statistical significance of testing the null hypothesis that there exists no difference in the mean values of each performance measure between our method and each of the other methods.

**Table 5:** Results of statistical analysis (*p*-values)

Classifier	Accuracy	Precision (PET)	Recall (PET)	F1 (PET)	ROC/AUC
Logistic Regression	2.52 × 10^− 08^	1.85 × 10^−09^	5.48 × 10^− 09^	5.87 × 10^−10^	1.46 × 10^− 09^
Decision Tree	1.80 × 10^− 04^	1.51 × 10^− 04^	6.99 × 10^− 06^	1.92 × 10^− 06^	1.16 × 10^− 05^
KNN	8.08 × 10^− 05^	6.22 × 10^− 05^	**1.40 × 10** ^**− 03**^	8.50 × 10^− 05^	**1.29 × 10** ^**− 04**^
SVM	1.17 × 10^− 08^	4.61 × 10^− 08^	1.74 × 10^− 04^	7.89 × 10^− 07^	5.02 × 10^− 06^
BoW + Logistic Regr.	**4.26 × 10** ^**− 04**^	**2.22 × 10** ^**− 04**^	1.79 × 10^− 04^	**9.85 × 10** ^**− 05**^	2.12 × 10^− 05^

The highest values are in boldface

## Discussions

As can been seen in Table 4, our word embedding + LSTM approach recorded highest means in 10-fold cross-validations in each and every performance measure listed, and *p*-values shown in Table 5 confirm that the difference in the mean values of each performance measure between our approach and each of other methods is of statistical significance with all *p*-values being less than 0.01 (*p* < 0.01). In other words, our word embedding + LSTM method demonstrates better performance than each and every other method investigated.

One can notice that the precision of predicting PETs has improved noticeably. Achieving higher precision of predicting positives is a much desired goal because higher precision results in more true positive and fewer false positive instances in the predicted positive (PET) class. Another related desired goal is to have a higher recall (or sensitivity), and is achievable by our method as evidenced in our results. A higher recall will help correctly identify more true positives and fewer false negatives from the data. Having higher accuracy, a measurement based upon prediction of both positive and negative classes, is important, but given the class-imbalance of the data which have more negatives, higher accuracy could be partially contributed by the imbalance. Therefore, accuracy is not our most importance concern.

While it is not clear to us why our method performs better than other methods, authors guess that word embedding along with the LSTM classifiers may extract richer semantic information from various expressions in tweet text which describe personal health experience, resulting in better classification performances. In our results, the adjustment of the class weight contributed to the improvement of performance measures, particularly recall, f-measure and ROC/AUC, in comparison with the results of our method without the adjustment (data not shown).

Finally, the word embedding-based vector space model was learned from the unlabeled tweets, significantly reducing the costly and lengthy annotation effort. We believe that this word embedding technique can help accelerate and scale up processing and classifying Twitter and any other text-based social media data, because the laborious tasks of engineering features are not required and unlabeled data can be used for feature learning.

## Conclusion

In this study, we investigated an approach of combining word embedding and neural network-based deep learning to identify tweets pertaining to the experiences related to health issues, in particular the tweets with experiences related to the consumption of pharmaceutical products. The outcome of our research demonstrates that our method outperforms, with statistical significance, other conventional algorithms in identifying personal health experience tweets. For health surveillance using social media data not involving feature engineering can help significantly accelerate and scale up the processing and analyses of the free text social media data with creatively shortened words or phrases and without following the grammar of a particular language.

## Abbreviations

API: Application programming interface
BoW: Bag of words
DNN: Deep neural network
LSTM: Long short-term memory, short for a type of neural networks which have “memory”
NLP: Natural language processing
PET: Personal experience tweet
POS: Part of speech
RNN: Recurrent neural network
ROC/AUC: Receiver operating characteristic/area under curve
VSM: Vector space model
